# Supplementary material for: Healthcare applications of single camera markerless motion capture: a scoping review
Source: PeerJ. 2022 May 26;10:e13517. doi: 10.7717/peerj.13517 (PMC9148557; doi:10.7717/peerj.13517)
Supplement: Supplemental Information 1 [file peerj-10-13517-s001.docx]

Supplementary 1: Healthcare Applications

|  | | | International Classification of Functioning, Disability and Health (ICF) | |  |  |
| --- | --- | --- | --- | --- | --- | --- |
| Reference | Target Population | Study Population | Measurements of Body Structure and Function (BCF) | Activity | Clinical Outcome | Primary Aim |
| (Tsuji, Nakashima & Hayashi, 2020) | Infants  Cerebral Palsy | 19 infants including low birth weight | - Movement magnitude - Movement balance - Movement rhythm - Movement of the body centre | Lying supine | General movement assessment (Prechtl’s) | Classify infants’ spontaneous movements into normal and abnormal movement based on Prechtl’s general movement assessment |
| (Schroeder, Hesse & Weinberger, 2020) | Infants  Cerebral Palsy | 29 infants (mean age 14.8 weeks) | - Fidgety movements | Lying supine | General movement assessment (Hadders-Algra) | - Evaluate movements represented by 3D body model of infant by performing GMA on 3d model and RGB video of same movements - Prediction of cerebral palsy using model and RGB were also performed and compared with follow up diagnosis |
| (Orlandi et al., 2015) | Infants  Cerebral Palsy | 3 healthy infants (10 days – 24 weeks after birth) | Tracking right-hand (RH), left hand (LH), right foot (RF) and left foot (LF):   - amplitude - average speed - acceleration | Lying supine | Not reported | Semi-automatic analysis of movement from video clip to support human observation by proving key movements of interest and trajectories |
| (Rahmati et al., 2016) | Infants  Cerebral Palsy | 78 (10 – 18 weeks):  14 with confirmed CP diagnosis | Movement trajectories | Lying supine | Not reported | - Using video motion data (specifically frequency variables) to predict cerebral palsy early - Comparison with marker-based method |
| (Orlandi et al., 2018) | Infants  Cerebral Palsy | 127 (3 – 5 months):   - 30 with musculoskeletal injury - 16 cerebral palsy | Movement tracking and velocity parameters were measured | Lying supine | General Movement Assessment (GMA) | - Parameters were used to classify typical and atypical general movements - Classify identification of infants with a diagnosis of CP (CP vs. not-CP) - Results were compared against clinical GMA |
| (Marchi, Hakala & Knight, 2019) | Infants  cerebral palsy | 21 (8 – 17 weeks):   - 7 later diagnosed with cerebral palsy - 14 with low neurodevelopmental risk | Keypoints | Lying supine | General Movement Assessment (GMA) | - Skeletal reconstruction of general movements was created using pose parameters - GMA was carried out on skeletal reconstruction and results compared with GMA of same raw videos to evaluate presence or absence of fidgety movements |
| (Støen et al., 2017) | Infants  Cerebral Palsy | 150 (10 – 15 weeks post term) | General Movements | Lying supine | General Movement Assessment (GMA) | Comparing results with clinical General movement assessment (GMA) |
| (Adde et al., 2010) | Infants  Cerebral palsy | 30 (10 – 15 weeks post term) with high risk of developing Cerebral Palsy:  13 later diagnosed with cerebral palsy | - Quality of motion - Velocity of motion - Acceleration of motion | Lying supine | Gross Motor Function Classification System (GMFCS) | - Movements were used to predict if infant would later be diagnosed with CP - Prediction also of prediction of ambulatory versus non ambulatory CP - CP predictions were compared with later CP diagnosis from clinician who used Gross Motor Function Classification System (GMFCS) |
| (Rahmati et al., 2014) | Infants  Cerebral palsy | 78 (10 – 18 weeks post term):  14 later diagnosed with CP | Motion trajectories of the following:   - Left ankle - Chest - Right wrist - Left wrist - Right ankle - Head | Lying supine | Not reported | - Classifying impaired vs unimpaired infant by assessing fluent vs non-fluent motion patterns - Results were compared with marker-based approach |
| (Groos et al., 2022) | Infants  Cerebral Palsy | 1424 recordings of post-term infants (9 – 18 weeks old) | Keypoints | Lying supine | General movement assessment (GMA) | Create an efficient markerless infant pose estimation method with comparable performance to human expert annotations |
| (Stahl et al., 2012) | Infants  Cerebral palsy | 82 infants (10 – 18 weeks post term):  15 confirmed diagnosed with CP by 5 years old | Fidgety movements:   - Absolute motion distance - Relative frequency - Magnitude of the wavelet coefficients | Lying supine | Not reported | Cerebral palsy classification is made based on calculated parameters to predict if subjects will later be diagnosed with cerebral palsy |
| (Reich et al., 2021) | Infants  Cerebral Palsy | 51 new-borns | Keypoints | Lying supine | General movement assessment (GMA) | Discriminating between fidgety movements and non-fidgety general movements |
| (Kidziński et al., 2020) | Cerebral Palsy | 1026 (Average patient age was 11 years (standard deviation, 5.9) with cerebral palsy | - Visit-level average walking speed - Cadence - Knee flexion angle at maximum extension | Gait | - Gross Motor Function Classification System (GMFCS) - Gait Deviation Index (GDI) | Parameters collected were used to predict the Gross Motor Function Classification System (GMFCS) score, and Gait Deviation Index (GDI) |
| (Chang, Han & Tsai, 2013) | Cerebral Palsy | 2 (both 14 years) | - Tracking 1 Dof of elbows - Tracking 2 Dof of shoulders - Number of correct movements | Rehabilitation exercise game performing upper limb tasks | Not reported | Parameters collected used to quantity number of correct movements during rehabilitation game with aim of providing motivation in rehabilitation |
| (Lee, Sinclair & Jones, 2019) | Parkinson’s | 8 with levodopa-responsive PD (44– 60 years) | - Amplitude - Velocity - Frequency | - Wrist pronation/supination - Hand Open/Close - Finger Tapping | Movement Disorder Society’s Unified Parkinson Disease Rating Scale (MDS-UPDRS) | Comparison of bradykinesia severity using parameters collected against (MDS-UPDRS) |
| (Grunert, Krause & Feig, 2019) | Parkinson’s | 15 with Parkinson’s (71.0 +- 10.4 years) | Not reported | Upper-limb rehabilitation game that simulates the throw of a basketball | Not reported | The focus is on correcting the symptoms of freezing and pisa syndrome |
| (Rocha et al., 2014) | Parkinson’s | 6 (46 – 59 years):   - 3 PD patients with implanted DBS stimulator - 3 healthy controls | - Velocity of the left/right foot, ankle, knee and hip, right/left hand, wrist, elbow and shoulder, central hip and shoulder, spine, and head - Acceleration of the left/right foot, ankle, knee and hip, right/left hand, wrist, elbow and shoulder, central hip and shoulder, spine, and head - Distance between feet, ankles, knees, hands, wrists, and elbows - Angle at left/right knee, right/left elbow, center shoulder, and spine - Stride duration - Cadence | Gait | Not reported | Discriminating between PD and non-PD subjects, and between Simulator on and off Patients |
| (Oña et al., 2018) | Parkinson’s | 5 with Parkinson’s (45 – 72 years) | - Joint trajectories - Movement ranges during exercises - Time taken to perform game | Rehabilitation games using the following activities: palmar prehension, finger flexion, and extension or hand pronation-supination | - Jamar handgrip dynamometer - Box and Blocks Test (BBT) - Purdue pegboard test | Feasibility of games to improve muscular strength, coordination, fine motor skills, and upper limb functionality of the upper limb in people with PD was assessed using performance on clinical outcomes before and after games |
| (Butt et al., 2017) | Parkinson’s | 28:   - 12 healthy controls (mean ± SD: 64.8 ± 9.1 years old) - 16 Parkinson’s (mean ± SD: 67.9 ± 6.9 years old) | - Number of rotational movements - Supination speed - Pronation speed - Variability of frequency - Variability of amplitude - Number of opening/closing movements - Hand opening speed - Hand closing speed - Variability of frequency - Variability of amplitude - Number of thumb-forefinger taps - Opening speed - Closing speed - Variability of frequency - Variability of amplitude - Signal strength of the movement - Relative power in the band of interest of postural tremor (8-12 Hz) | - Pronation/supination of the forearms (PSUP) - Opening/closing of the hands (OPCL) - Thumb-forefinger tapping (THFF) - Postural tremor (POST) | MDS/UPDRS III | - Classify between healthy and Parkinson’s subjects - Comparing results with clinical scores from MDS/UPDRS III |
| (Rocha et al., 2015) | Parkinson’s | 9:   - 5 healthy controls (54 +- 16 years) - 4 PD patients treated with Deep brain stimulation (62 +- 3 years) | - Velocity of the left/right foot, ankle, knee and hip, right/left hand, wrist, elbow and shoulder, central hip and shoulder, spine, and head - Acceleration of the left/right foot, ankle, knee and hip, right/left hand, wrist, elbow and shoulder, central hip and shoulder, spine, and head - Distance between feet, ankles, knees, hands, wrists, and elbows - Angle at left/right knee, right/left elbow, center shoulder, and spine - Stride duration - Cadence | Gait | Not reported | Compare Kinect v1 with Kinect v2 when discriminating between PD and non-PD subjects, and stimulation on and off subjects. |
| (Palacios-Navarro, García-Magariño & Ramos-Lorente, 2015) | Parkinson’s | 7 with PD (67 – 72 years) | Not reported | Rehabilitation game involving lower limb movements | 10 meters walk test (10MWT) | Efficacy of game was tested by measuring improvements in completion time score and in 10 meters walk test score carried out by clinician before and after game |
| (Sato et al., 2019) | Parkinson’s | 119:   - 117 HC (: “mostly in their 20’s”) - 2 with PD (60,70 years) | Cadence (steps/min) | Gait | Not reported | Calculate cadence from video filmed in a hospital clinical setting |
| (Galna et al., 2014) | Parkinson’s | 19:   - 9 with PD (mean: 68.2y) - 10 HC (mean 27.5 y) | - Sit to stand (mm) - Standing trunk flexion (deg) - Lateral trunk flexion (deg) - Forward stepping (deg) - Side stepping (deg) - Shoulder flexion (deg) - Shoulder abduction (deg) - Elbow flexion (deg) - Hand clasping (mm) - Pronation supination (mm) - Leg agility (mm) - Walking on the spot (mm) | - Quiet standing - Multidirectional reaching and stepping and walking on the spot - Hand clasping - Finger tapping - foot, leg agility - Chair rising - Hand pronation | Unified Parkinson’s Disease Rating Scale | Comparing results from Kinect with gold standard Vicon system |
| (Eltoukhy et al., 2017) | Parkinson’s | 19:   - 11 hc (71.1 += 7.5 y) - 8 PD (71.0 +- 5.6 y) | - Step length (m) - Step width (m) - Mediolateral pelvis displacement (m) - Vertical pelvis displacement (m) - Step time (s) - Stride time (s) - Foot swing velocity (m/s) - Ankle rom - Knee rom - Hip tom - Trunk rom | Gait | Not reported | - Comparing with gold standard Vicon system - Measure differences between control and PD gait patterns |
| (Latorre et al., 2018) | Stroke | 83 (18-80 years):   - 45 HC - 38 post-stroke | - Speed (m/s) - Stride length (m) - Stride time (s) - Step length (m) - Step time (s) - Step asymmetry - (m) - Double support - time (s) - Swing time (s) | Gait | Not reported | Estimate spatiotemporal gait parameters using the Kinect v2 against measurements estimated by visual inspection of video camera recording |
| (Bonnechère et al., 2018) | Stroke | 43:   - 16 healthy adults (age not reported) - 17 elderly (79+-5 years old) - 10 patients with chronic stroke (73 +- 8) | Upper-limb joints (right shoulder, left shoulder, right wrist, left wrist):   - Length (mm) - Angle (deg) - Velocity (m/s) - Angular velocity (deg/s) - Volume (mm3) - Sphere (cm3) - Surface (mm2) | Rehabilitation game | Not reported | - Validate results from markerless against marker-based system - Provide joint trajectory visual analysis for easy comparison between patients and patient follow up |
| (Scano et al., 2014) | Stroke | 5 (25 – 70 years):   - 4 healthy - 1 neurological patient | Kinematics:   - Shoulder elevation angle (SA) at full extension - Elbow flexo/extension angle (EA) at full extension   Dynamics:   - Shoulder elevation torque at full extension - Effort Index - Motor Control: - Coefficient of Periodicity - Normalized jerk (NJ) index | Reaching against gravity (RCH) movements | Not reported | Compare the performances of a passive-marker motion capture system  with the Kinect for use in assessing upper-limb functionality at home |
| (Vernon, Paterson & Bower, 2015) | Stroke | 30 post-stroke (68 ± 15) | - Trunk flexion (deg) - Flexion angle velocity (deg/s) - Step length (m) - Stride length (m) - Gait speed (m/s) - Turning time (s) - Total time (s) | Time up and go test (TUG) | - Timed Up and go - 10-meter walk test | - Test reliability of Kinect measured TUG variables against clinical assessment of variables - TUG parameters were used to predict outcome on 10MWT, TUG, Step Test (ST), and Functional Reach (FR) and compared with test carried out by clinician |
| (Ozturk et al., 2016) | Stroke | 5 (55 – 61 years):   - 3 Stroke - 2 healthy controls | - Joint trajectories - Speed profile of movement - Wrist speed - Straightness - Shoulder Range of motion - Elbow range of motion - Spectral Arc-length - Maximum Speed - Index of Curvature - Trunk Displacement, - IJCI: Inter-joint coordination index | Reaching task | Wolf Motor Function Test (WMFT) | Comparing results with Wolf Motor Function Test (WMFT) |
| (Behrens, S & T., 2016) | Multiple Sclerosis | 149:   - 90 MS - 59 HC | - Body’s centre of mass - Body sway - Mean angular sway velocity - Mediolateral direction | Three static stances: open stance, closed stance and tandem stance | - SMSW - T25FW - MSWS-12 | - Assess postural control by calculating the 3D mean angular sway velocity (MSV-3D) - Association of MSV with clinical outcomes |
| (Behrens et al., 2014) | Multiple Sclerosis | 44:   - 22 healthy controls (37 += 11 years) - 22 MS (mean +- SD 43 +- 9 years) | Average walking speed (m/s) | Gait | - Short Maximum Speed Walk test (SMSW) - The Expanded Disability Status Scale (EDSS) - The Timed 25-Foot Walk (T25FW) | - Established new digital and observer independent measure Short Maximum Speed Walk test (SMSW) to detect walking speed using hip joint centre tracking - SMSW was compared against clinical outcome measures |
| (Grobelny, Behrens & S., 2017) | Multiple sclerosis | 145:   - 95 with MS - 60 HC | - Trajectory of hip center joint - Average speed (m/s) - Speed deviation (m/s) - Mediolateral deviation (cm) - Vertical deviation (cm) - 3D deviation (cm2) | Gait | - Short Maximum Speed Walk (SMSW) - 25ft walk (T25FW) | - Assess association of SMSW derived parameters with clinical disability and gait impairment - Equivalence of SMSW to rater-based clinical outcomes |
| (Rémi et al., 2011) | Epilepsy | 17 | - Wrist movement extend - Trunk speed - Wrist speed - Trajectories of body, trunk center and left and right wrists | Seizure | Not reported | Classify seizures into hyperkinetic or automotor |
| (Pereira Choupina et al., 2018) | Epilepsy | 111 with seizures (age not reported) | Seizure movement | Lying in The Early Assessment Medical Unit (EMU) bed | Not reported | Seizure movement data is used to classify seizure syndrome and count number of seizures |
| (Lange et al., 2011) | Neurological Injury:   - Balance Issues related to stroke - Traumatic brain injury - Spinal cord injury | 20 (age not reported):   - 10 with balance issues related to stroke - 3 with traumatic brain injury - Spinal cord injury | - limit of stability - arm length | Rehabilitation Game | Not reported | Create a tool for balance training of adults with neurological injury using collected parameters to change game difficulty to appropriate level |
| (Chen et al., 2016) | Essential tremor (ET) | 4 with ET (69 – 71 years) | - Trajectory of fingers - Tremor amplitude - Tremor frequency | Upper limb reaching task | Not reported | Measure severity of finger tremor using tremor amplitude |
| (Nakamura et al., 2019) | Cervical dystonia | 30 with cervical dystonia (average 52.3±16.0 years) | - Angles of the yaw axis (rotation) - Roll axis (lateral tilting) - Pitch axis (sagittal flexion and extension) | - Rotation - Laterocollis - Antecollis/retrocollis | Toronto Western Spasmodic Torticollis Scale (TWSTRS) | Semi-automated scoring system for the Toronto Western Spasmodic Torticollis Scale (TWSTRS) severity scale |
| (Mehdizadeh et al., 2021) | Dementia | 54 | - Step time (s/week) - Step length (cm/week) - Step time CV (%/week) Step length CV (%/week) - Sacrum ML velocity RMS (cm/s) - Gait speed (cm/s/week) - Step width (cm/week) Step width CV (%/week) | Gait | Fall risk characteristics such as demographic data, geriatric psychiatric assessment and history of falls | Describe changes in gait over 10 weeks in hospitalised to understand gait deterioration and fall risk |
| (Capecci, Ceravolo & Ferracuti, 2018) | Chronic disabilities due to neurological (Parkinsons, stroke) and musculoskeletal  disorders (backpain) | 57:   - 28 Healthy controls (22 – 76 years) - 29 Experimental group (17 – 76 years) | Primary outcomes:   - LMin and LMax of underarm angle in the sagittal plane - LMin and LMax of the lateral shoulder flexion in the frontal plane respect to hip - LMin of the horizontal distance between elbows - LMin and LMax of the spine base oscillation in the transverse plane - LMin and Lmax of the knee angles in the sagittal plane   Control Factors:   - Elbow extension angles - Knee extension angles - Hip angles - Torso Area - Hands Distance - Ankle Distance | 5 exercises widely used for physiotherapy of axial disorders | Not reported | Assessing motor performance during rehabilitation by providing a quantitative score for each Primary outcome and Control factor |
| (Carmo Vilas-Boas et al., 2019) | Transthyretin Familial Amyloid  Polyneuropathy (TTR-FAP) | 6 with Transthyretin Familial Amyloid  Polyneuropathy (TTR-FAP) (34 – 52 years) | - Stride duration (s) - Stride length (cm) - Step duration (s) - Step length (cm) - Gait speed (m/s) - Gait speed variability (m/s) - Spine shoulder angle (deg.) - Spine middle angle (deg.) Maximum elbow angle (deg.) - Minimum elbow angle (deg.) - Maximum knee angle (deg.) - Minimum knee angle (deg.) - Ankle angle range (deg.) | Gait | Not reported | Compare parameters collected using Kinect against reference motion capture system (Qualisys) |
| (Vilas-Boas et al., 2019) | Transthyretin Familial Amyloid Polyneuropathy (TTR-FAP) | 10 with TTR-FAP (Mean 36 +- 7 years) | Spatiotemporal:   - Stride duration - Step duration - Stance duration - Swing duration - Single support duration - Double support duration - Stride length - Step length - Step width - Gait speed - Gait speed variability - Foot swing velocity - Arm swing velocity - TBCM sway x/y-component   Kinematic   - Spine shoulder angle - Spine middle angle - Maximum elbow angle - Minimum elbow angle - Maximum knee angle - Minimum knee angle - Hip angle range - Ankle angle range | Gait | Not reported | Evaluates the agreement between the Kinect v2 and a reference Qualisys system for each parameter |
| (Vilas-Boas et al., 2020) | Hereditary amyloidosis associated with transthyretin V30M (ATTRv V30M) | 66:   - 25 Healthy Control (HC) – (19 - 51 years) - 14 asymptomatic ATTRv V30M carriers (AC) (23 – 54 years) - 27 Symptomatic   ATTRv V30M carriers (SP) (23 – 63 years) | - Stride duration, s - Stride length, cm - Step duration, s - Step length, cm - Step width, cm - Stance duration, s - Swing duration, s - Single support duration, s - Double support duration, s - Gait speed, m/s - Gait speed variability, m/s - Foot swing velocity, m/s - Arm swing velocity, m/s - Total body center of mass sway in x-axis, mm - Total body center of mass sway in y-axis, mm - Neck angle, deg - Spine shoulder angle, deg - Spine middle angle, deg - Maximum elbow angle, deg - Minimum elbow angle, deg - Maximum knee angle, deg - Minimum knee angle, deg - Hip angle range, deg - Ankle angle range, deg | Gait | Not reported | Distinguishing different disease stages based on collected gait parameters |
| (Han et al., 2015) | Facioscapulohumeral muscular dystrophy (FSHD) | 44:   - 22 with fshd (52.5 +- 19.0 years) - 22 healthy controls (: 44.6 += 15 years) | - Upper-limb trajectories - 4 Quadrants of reachable space surface area - Absolute total reachable workspace surface - Envelope area (m2) - Arm length | Upper limb reaching movements | FSHD evaluation scale | - Validate new outcome measure to determine the spectrum of reachable workspace encountered in FSHD by calculating mean relative surface area (RSA) for each quadrant |
| (Han, Bie & Nicorici, 2016) | Duchenne muscular dystrophy | 53:   - 29 with DMD - 24 healthy controls | - Upper-limb trajectories - 4 Quadrants of reachable space surface area - Absolute total reachable workspace surface - Envelope area (m2) - Arm length | Upper limb reaching movements | Upper limb (PUL) assessment | Compare reachable workspace measure with PUL assessment by clinician |
| (Stone & Passive, 2012) | Elderly | 7 elderly (75 – 95 years) | - Average speed (cm/s) - Average stride time (cm/s) - Average stride length (cm) - Height (cm) | Gait | Not reported | Measurements were used to classify number of quality walks over 21 days |
| (Stone & Skubic, 2013) | Elderly | 17 elderly (68 – 98 years) | - Walking speed (cm/s) - Height (cm) | Gait | - In-home gait speed (IGS) - Timed up and g time (TUG) | IGS was calculated using collected parameters and used to estimate TUG |
| (Stone & Skubic, 2015) | Elderly | 16 elderly (69 to 97 years) | Vertical state and on ground event segmentation of fall motion | Gait | Not reported | Fall detection |
| (Dolatabadi, Taati & Mihailidis, 2014) | Total Hip Replacement (THR) on right side | 1 with THR surgery  64 years | - Step Length - Stance Time - Cadence - Angular velocity of hip flexion - Horizontal velocity of center of mass - Velocity of center of mass - Angular velocity of hip extension | - Gait - Sit to stand tasks (STS) | Not reported | Monitor changes in movement patterns before and after a total hip replacement surgery |
| (Lee, Yoon & Chung, 2015) | Adhesive capsulitis of the shoulder (AC) | 27:   - 15 HC (45+-9 years) - 12 with AC (52 +- 9 years) | - Range of motion of affected shoulders - Flexion angle of the shoulder - Abduction angle of the shoulder - Rotational angle of the shoulder - The peak angles of each repeated motion were averaged to determine the ROM angle in that direction | Shoulder flexion, abduction, and external rotation as the subject was standing | Goniometer | - Validate Kinect measurements by comparing against goniometer |
| (Capecci, Ceravolo & Ferracuti, 2016) | Motor disabilities due to different unreported pathologies | 33:   - 19 healthy controls (22 – 31 years) - 14 with unreported motor disabilities due to different pathologies (42 – 72 years) | - Range of motion - Motion trajectories | Rehabilitation game using 5 exercises widely used for low back pain physiotherapy | Not reported | Compare hidden semi-Markov model and dynamic time warping to understand which method best correlates with clinical evaluation of exercises |
